# Proceedings of a workshop, held in Constanta, Romania on 22 May 2014, on Oral Health of Children in the Central and Eastern European Countries in the context of the current economic crisis

**DOI:** 10.1186/s12903-016-0223-y

**Published:** 2016-07-25

**Authors:** Dorjan Hysi, Kenneth A. Eaton, George Tsakos, Paula Vassallo, Corneliu Amariei, the DPH Group

**Affiliations:** 1 Albanian Dental Association, University Dental Clinic, Tirana, Albania; 2 European Platform for a Better Oral Health in Europe, Old Saddlers, Canterbury Road, Ashford, London, Kent TN25 4EW United Kingdom; 3 European Association for Dental Public Health, London, United Kingdom; 4 Council of European Chief Dental Officers, Valletta, Malta; 5 Romanian Association of Oro-Dental Public Health, Constanta, Romania

**Keywords:** Oral health, Central and Eastern Europe, Economic Crisis, Workshop, Proceedings

## Abstract

This report presents the proceedings of a workshop held in Constanta, Romania on 22 May 2014. During the workshop, representatives from 18 Central and Eastern European countries gave oral presentations on the current oral health of children and young adults aged 16 years and younger. The aim of the workshop was to collect and present data relating to the oral health of children from Central and Eastern European countries and to discuss them in the context of the political changes that have taken place over the last two decades and the recent economic crisis.

The presenters had previously completed a series of questions on oral epidemiological studies, prevention of oral disease, treatment and payment, dental personnel, uptake of oral health care and other considerations and structured their presentations on these topics plus the influence of the economic crisis on oral health. It should be remembered that this paper is a report of the proceedings of a workshop and not a study. Ethics approval is not required for workshops.

After the 18 oral presentations a 90 min discussion took place during which further points were raised. The presentations, the discussion and the conclusions which were reached are reported in this manuscript.

## Background

Oral health is an integral and very important part of general health [1]. Diseases originating from the mouth can effect general health and vice versa. There is documentation in the literature about prevalence of and trends in oral diseases in most of the western European countries [2, 3], where, in general, especially in North West Europe, surveillance studies seem to be well organized for collecting, analyzing and interpreting the data for oral health planning. These countries have been democracies for a long time. This might have influenced positively the oral health care systems, resulting in a reduction the prevalence of dental caries in children and young adults and an increasingly dentate elderly population. In contrast, oral health data are scarce in many Central and Eastern European (CEE) countries. There is relatively little in the literature from these countries about oral diseases prevalence, preventive programs and systems for the delivery of oral care. Furthermore, when presenting such epidemiological data, either verbally or in writing, some speakers/authors may be unable to cite appropriate references.

Since, 1990, the CEE countries have undergone political and economic changes that have affected not just their health care systems but also their lifestyles and their attempts to reach European Union (EU) standards. Some of the CEE countries have joined the EU, others are in the process and others have decided not to apply. The oral health care systems of these countries are very diverse. Since 2008, the global economic crisis has led to a general perception that budget cuts have affected all parts of the economy including general and oral health care in these countries. Because many of the citizens of CEE countries are impecunious or very impecunious, the combined effects of the economic crisis and a change in some countries, from a centralized economy to a market economy, has led to a privatization of oral care services. This has potentially created a cost barrier to seeking oral health care and, as a result, a deterioration in the oral health of many citizens of these countries.

Against this background, a workshop was planned and organized by the European Association of Dental Public Health (EADPH) and the Romanian Association of Oro-Dental Public Health. It was sponsored through the generosity of the Borrow Foundation.

## The aim of the workshop

The aim of the workshop was to collect and present data relating to children’s oral health from Central and Eastern European (CEE) countries and to discuss them in the context of the political changes that have taken place over the last two decades and the recent economic crisis.

## Objectives of the workshop

Within the aim there were a number of objectives, which were to:

- Gather the most recent epidemiological data on the oral health of children.
- Ascertain if there were any current or past national, regional or local programmes to prevent oral diseases in children.
- Understand the payment systems for the oral health care of children
- Ascertain the structure of the oral health workforce
- Ascertain the uptake of oral health care by children
- Understand the affect of the economic crisis on the provision of oral health care for children

## Organization of the workshop and participants

The workshop was organized by Professor Corneliu Amariei (President of Rumanian Association of Dental Public Health) and Professor Kenneth Eaton (Chair of the European Platform for Better Oral Health in Europe). Its sessions were moderated by Dr Paula Vassalo, President of Council of European Chief Dental Officers, Dr Georgios Tsakos, President of the EADPH and Professor Eaton.

There were 18 invited participants who came from Dental Faculties, Dental Associations and Ministries of Health in Albania, Armenia, Belarus, Bulgaria, Croatia, Czech Republic, Georgia, Greece, Hungary, Latvia, Lithuania, Macedonia, Moldova, Romania, Russia, Slovakia, Turkey and Ukraine. Unfortunately, the delegate from Armenia was unable to attend the workshop. However, he submitted a report which was read on his behalf. The names of the 18 invited participants who presented data from their countries and wrote the reports that appear in the appendix to this report are in Table 1.

It is difficult to define what constitutes Central and what constitutes Eastern Europe. Prior to 1914, Central Europe was considered by many to consist of the countries of the Austro-Hungarian Empire. However, after 1918 and again after 1945 borders were redrawn. In the early 1990s further changes occurred with the break-up of Former Yugoslavia into its constituent countries and the division of Czechoslovakia into two countries. Thus, there appears to be no clear definition of what should be considered as Central and Eastern Europe. Perhaps, of the countries who attended the workshop, those from the former Soviet Union plus Turkey could be considered to be Eastern European and the other countries as Central European. The choice of which countries were invited to take part in the workshop was relatively arbitrary and was made on the basis of the organisers’ acquaintance with senior academics and advisers to Health Ministries in the countries concerned. Representatives from all 18 countries that were invited accepted the invitation and produced reports. However, as previously stated, the representative from Armenia was unable to attend in person and his report was read on his behalf. Nine of the countries represented were Member States of the European Union (EU). They were Bulgaria, Croatia, Czech Republic, Greece, Hungary, Latvia, Lithuania, Romania, Slovakia, The other nine were not members of the EU and only three (Albania, Macedonia and Turkey) were at the time actively seeking membership of the EU. With the exception of Poland, representatives from all CEE countries with a population of more that 10 million took part in the workshop. The total population of the 18 countries is over 80 % of the total population of all CEE countries.

In January 2014, a questionnaire (Fig. 1) was prepared and sent to all delegates, who completed it and sent their answers to the workshop organisers. Their responses were published in the workshop program in advance of the workshop. The questions were structured to focus delegates on specific topics and to try to obtain comparable data. This was not a study but a workshop so ethics approval was not required.

The participants were welcomed by Professors Eaton and Amariei, who explained the aim of the workshop and the program. There were two presentation sessions followed by the discussion of the working group, a plenary discussion and conclusions.

Each country’s representative gave a 10 min presentation which focused on the points raised in the questionnaire i.e. epidemiological data, prevention, treatment and payment, dental personnel, uptake of oral health care and other considerations. The presenters also authored the descriptions from each of the 18 CEE countries; these appear in the appendix to this report.

## Summary of the presentations and discussion

This section of the proceedings summarizes the presentations and the discussion that took place after the presentations. Where possible, the data that were presented are summarised in tables for the topics concerned.

### Epidemiology (Table 2)

Presenters introduced data from their national or local studies. Most of them focused on dental caries prevalence of 5 and 12 years old. In general, the WHO 1997 criteria were used and caries was recorded at D_3_ level, by caries prevalence or DMFT/dmft indices. The data indicated that high dental caries prevalence was still the main dental public health problem in most of the countries represented at the workshop. The national mean DMFTs for 12-year-olds, which were presented during the workshop, were higher in the Eastern European countries (between 2.05 and 6.77) compared with the Western European countries (between 0.6 and 1.70) [4]. However, the variability in the methods used and the wide range of years in which the studies took place made it difficult to compare them. The methodologies used to gather the data were not consistent and comparable. Some of the studies were local and did not represent a national sample. Also training, calibration and sample selection criteria were questionable in some of the studies. For these reasons it is unwise to compare these data between countries and therefore no tables to show the different reported dmft and DMFT data are presented in this report.

Only a few data were presented about periodontal status, the need for orthodontic treatment and oral cleanliness. There was no provision for periodic national surveys to monitor oral diseases in any of the 18 countries, whose representatives attended the workshop. As mentioned in the introduction, some presenters were unable to cite references to support the data that they presented and have subsequently been unable, or unwilling to do so during the production of this report.

### Prevention (Table 3)

Prevention programs which focused on dental caries were more frequently at a local rather than a national level. During the discussion, after the formal presentations, it became apparent that Romania and Bulgaria encourage milk fluoridation programs in some parts of their countries. The representative from Latvia presented a national preventive program that was very well designed and implemented. Until 2010, the Latvian program achieved a nearly 50 % reduction in the national mean DMFT score of 12 -year-olds. It consisted of several components including education, fissure sealing and the use of fluoride varnish. Most of the countries did not have preventive programs at a national level. There was a general perception that there was a need to put more effort in prevention by the health authorities (Ministries and local government), academia, the European Commission and international foundations.

### Treatment and payment

Some countries indicated that free treatment was provided by their dental public services for the age group 0–16 years and it included fissure sealing, fluoridation, scaling and polishing, extractions and fillings. In Croatia there was a comprehensive free service for children which included prosthetic and orthodontic treatment. Treatment in the private dental service was not free except in countries where the state insurance scheme covered prevention and some other procedures e.g. in Macedonia. In other countries, parents and caregivers had to pay for all dental treatment provided in the private dental clinics/offices/practices. No one presented data concerning toothpaste sales and oral health expenditure.

### Dental personnel (Table 4)

Dental treatment for children was always performed by a dentist, except in Latvia where dental hygienists are an important part of the team. In most countries, general dentists were the main providers of oral health care. One clear exception was Georgia where every dental graduate underwent further education and became a specialist. In many of the 18 countries, dentists work solo and without chairside assistance. Only six countries - the Czech Republic, Hungary, Latvia, Russia, Slovakia and the Ukraine- train and employ dental hygienists. Many countries had declined to train dental hygienists because they had un- or under-employed dentists and were continuing to overproduce dentists. Some of the speakers expressed a fear that the opening of new dental faculties, often as private schools without state funding, was having a great impact and was leading to the graduation of too many dentists, who had been trained to a lower standard. Another concern expressed was the risk of overtreatment in countries where there are insufficient patients for the dentists. Speakers, from countries with this problem, indicated that the main reasons for this chaotic and “ridiculous” situation were a lack of workforce planning and inappropriate politics. In many of the 18 countries, access to oral healthcare was difficult in rural areas, since dentists tend to migrate to urban areas. The Latvian speaker explained that in her country this problem had been addressed by the use of mobile dental clinics which travel to remote rural areas. During the discussion it became apparent that only Latvia and Slovakia had data on migration of dentists to other countries.

### Other considerations

Many of the countries indicated that the economic crisis in the last 6 years had negatively influenced the provision of oral health care. There had been budget cuts and reductions in the public dental workforce responsible for oral health care of children. However, there were exceptions, such as in Croatia where there have been no cuts and all children and young people can still obtain all oral health care free of charge until they are 18 years old.

The situation does not seem to be improving. There was agreement on the need for a wide coalition to make oral health an important issue not only to the local and national governments but also the European Commission.

During the discussions, after the oral presentations, it was agreed that:

- There is a need to improve the quality of oral epidemiological data through better study design, applying consistent methodologies and, if the WHO oral health assessment criteria are used, to employ WHO 5^th^ edition Basic Methods for Oral Health Surveys [5]. In the future, the International Caries Detection and Assessment System (ICDAS) Epi version could be used.
- Special attention to training examiners in epidemiological surveys should be consistent and contribute to high levels of intra and inter examiner consistency.
- A simple questionnaire should be given to people who are subjects in epidemiological surveys to obtain a snapshot of the their awareness of the need for oral health care and their socio-economic status.
- There was a need for a workshop on epidemiology of oral health to minimize the inconsistencies and to improve their knowledge toward survey methodologies.

## Conclusions

The aim of the workshop had been to bring representatives from 18 CEE countries together to compare the current situation with regard to the oral health of children in their countries and the impact of the economic crisis on the provision of oral care.

The reports, which each representative produced, gave an overview of the situation in their country and enabled them to share good practice. The workshop provided a great opportunity to meet colleagues from CEE countries and discuss different experiences and problems. It seemed that all had common problems as well as some that were unique to individual countries. It is clear that a great deal of work needs to be done towards improving the prevention or oral diseases, oral epidemiological surveys and the systems for the provision of oral care in all the countries represented at the workshop.

All those who attended the workshop expressed their gratitude to the organizers, the EADPH and the Romanian Association of Dental Public Health, and to the Borrow foundation for their generous sponsorship, without which the workshop would not have taken place.

## Appendix

### Reports from the 18 Countries

#### Albania

**Epidemiology**

The last national study was conducted in 2011. Children aged 6 and 12 years were examined. Dental caries experience for the age group 6 and 12-year-olds was assessed. There has been no study measuring oral cleanliness, the need for orthodontics and traumatized anterior teeth.

**The data for the 12-year- olds**

The caries prevalence (DMFT > 0) was 87 %, 29.6 % were without cavitated carious lesions (DT = 0) [6].

The mean caries experience (DMFT) was 3.73 (SD 2.71) for boys, 3.71 (SD 2.61) for girls and 3.72 (SD 2.66) overall [7].

The caries treatment need DT/DMFT was 56 % (sd 0.36), the mean FT/DMFT (Care Index) was 31 % (SD 0.34) and the mean Significant caries index (SiC) was 6.72 (SD 1.92) [6]

**The data for the 6-year- olds**

Mean dft = 3.75 (+/- 3.2), SiC = 7.5 (+/- 2.3), The prevalence of caries free 6-year-olds was 20.5 % (dft = 0). The prevalence without cavitated lesions (dt = 0) for the whole group was 23.1 % and the caries treatment need was d/dft = 0.9 [7].

There has also been a local study in Tirana on the dental caries experience and oral health behaviour of 12- year-olds in this capital city [6]. The main results were: The mean DMFT was 3.8, DMFS =7.98, Significant Caries (SiC) index =7.06, and the prevalence of caries-free children was 14.5 %. The percentage of untreated caries or the ratio of D/DMFT was 0.542 (54.2 %) [8].

- 87 % participants reported consuming sweets every day.
- 67 % reported brushing their teeth at least once a day.
- 21 % had knowledge of dental floss.
- 67,2 % brushed their teeth every day.

The children reported making frequent visits to a dentist, often for relief of pain.

**Prevention**

There is no national preventive program for oral health diseases in Albania. There was a pilot study (program) of fluoride varnish application in a selected group of children (12-year- olds) in Tirana. There are oral health education programs in schools organized by local health authorities and publicly employed dentists. These programs do not cover all the country and are not well organized.

The programs take place in schools but not on a regular basis. In the private sector, dental cabinets (clinics) are used mainly for treating oral diseases.

**Treatment and Payment**

It is mandatory by law for the public service to offer free dental treatment to 0–18- year-olds. This free service covers preventive and restorative dentistry, endodontics and oral surgery. There is no free service for prosthetics and orthodontics. The free service is offered by public clinics only. There is no free service offered by private practitioners.

**Dental Personnel**

In Albania only general dentists or general dentists and specialists in children’s dentistry and orthodontists provide clinical treatment. Oral health care is provided by the public dentists who are mainly general dentists, there are a few specialists in children’s dentistry. Orthodontics is provided by specialists only at the University Dental Clinic in Tirana (public institution). Since 2009, there have been no dental nurses in the public service. There are dental nurses only at the University Dental Clinic in Tirana.

There are no official data for private practice but most dentists employ chair-side assistants who may be qualified nurses, or trained on the job personnel, or dental students or unemployed dentists after graduation. Dental Nurses perform chair-side assisting and sterilization.

There are no dental hygienists in Albania. There is an over production of new graduates (dentists) from dental schools (several private schools opened 5–10 years ago) and most of the graduates from these schools will perform dental hygienists’ work since there is not enough work for dentists (poor workforce planning).

**Uptake of Oral Health Care**

There are no national data. There are some data from a local study published in 2010 about the 12 year olds living in Tirana [5] They indicate that 67 % visited a dental office (cabinet/clinic) at least twice and for some, more over a two year period.

People living in rural areas, especially those far from the main towns, do not have access to dental services.

Provision of dental service is 95 % private and is located in the main urban areas. The public service is also located in the urban areas in dental clinics and schools.

**Other Considerations**

There has been a budget cut for public service resulting in less treatment, infrastructure and equipment maintenance and replacement and fewer public dental personnel.

As for the private dental service, the prices are not affordable for most of the population.

The situation seems to be the same, no better or worse as a result of the economic crisis

What is needed is a national preventive program that will coordinate the public and private service especially in rural areas. The Ministry of Health, Non-Governmental Organizations and other Foundations would need to support such a program.

Oral health education topics should be part of the primary and secondary school curriculum.

More emphasis should be given to the preventive curriculum in dental schools and continuing education and training organized by the public, private sector and dental associations.

Media involvement, to raise the profile of oral health, is very important.

#### Armenia

**Epidemiology**

The last survey was carried out in 2013.

Children up to the age of 14 years were examined.

The resulting data are still being analyzed, and have not been published yet.

**Prevention**

There is an annual program on prevention of oral diseases for 6 and 12 year-old children in Armenia. It is run on a national basis. The program takes place in middle schools.

**Treatment and Payment**

There is free oral treatment for all children up to the age of 16 years for all types of dentistry, extractions, fillings, crowns, scaling and polishing, orthodontics, fixed and removable prostheses, and additionally free orthodontic treatment for 12-year-old children.

There is free oral treatment for children up to the age of 18 years, if they are from some special social groups defined by the government. This is provided in public clinics and private clinics certified for free treatment by the Ministry of Health.

**Dental Personnel**

Oral health care for children is provided by specialists in children’s dentistry and orthodontists.

All dentists (100 %) employ dental nurses. Dental nurses only assist dentists at the chair-side and are not allowed to perform any procedure on patients.

There are no dental hygienists working in Armenia.

**Uptake of Oral Health Care**

Nationally in 2013, a total of 63,563 children aged 14 years or younger visited a dentist (in that year 585,020 Armenians were aged 14 years or younger).

In rural parts of country there are some difficulties in accessing dental care. This is due to a lack or absence of local dental offices (cabinets/clinics).

**Other Considerations**

Because of deterioration of the economic situation in Armenia, as in the rest of world, there are signs of reduction in the provision of oral health care.

As a consequence, there seems to have been a deterioration in the oral health of children up to the age of 16 years.

To improve the situation the organization and development of a public oral health care prophylactic and research program is necessary.

#### Belarus

**Epidemiology**

National caries prevalence surveys have been performed in 2008 [9] and in 2014 In both surveys 6, 12 and 15-year old children were examined in the five major cities of Belarus, with 300 for each age group in each city. The schools and children were chosen at random. The main results for the 2008 survey are shown in Tables 5 and 6.

There is no information about orthodontic needs and prevalence of 16 years old with traumatized anterior teeth.

**Prevention**

Between 1998 and 2013, there has been a national program for the prevention of dental caries and periodontal diseases in the Belarusian population. The next program for 2014–2022 is about to be confirmed.

- The program will run at a national, regional and local level and will take place in kindergartens, schools, dental offices (cabinets/clinics) and via the mass media.

**Treatment and Payment**

For children up to the age of 16 years old, dental treatment is free of charge except for orthodontics. This free treatment is provided in public clinics.

**Dental Personnel**

Oral health care is provided by children’s dentists and orthodontists.

100 % of dentists employ dental nurses to provide chair-side assistance. The dental nurses prepare the clinic, dental materials and instruments and sterilize all necessary equipment.

There are no dental hygienists working in Belarus.

**Uptake of Oral Health Care**

80 % of children age 16 years old or less visited a dentist in 2013.

There are no parts of the country where it is difficult or impossible to visit a dentist.

**Other Considerations**

In the last six years, more private dental practices have been established and higher fees are paid by patients for treatment.

The data indicate that the oral health of children seems to be improving. This may be due to the motivation and learning of children and their parents about the oral health care.

#### Bulgaria

**Epidemiology - (The Need for Oral Health Care)**

The last national study of the oral health of children was performed in 2010 [10]. Children and young people aged 5–6, 12 and 18 years old were examined**.** The resulting data are set out in the Tables 7 and 8.

No data on traumatized anterior teeth were collected during this survey.

**Prevention**

There was a national program for first molar fissure sealing from 2009 to 2014. It took place in dental offices (cabinets/clinics).

**Treatment and Payment**

For children aged 16 years and under the following treatments are performed free of charge: extractions, fillings, endodontics (up to two teeth each year). A total of one check up and four items per year are included in the free of charge plan.

These treatments are covered by the National Health Fund (NHF) budget. These services are provided by the private dentists contracted to the NHF [11].

**Personnel**

Only dentists provide dental treatment in Bulgaria and there are no dental hygienists working in the country. The lack of dental hygienists correlates with the oversupply of dentists [12].

About 40 % of dentists employ dental nurses. They assist them at the chair-side and “take care” of the clinic part of the dental office.

**Uptake of Oral Health Care**

There are no official data on uptake of oral care. Because of the large numbers of dentists, there are no parts of the country where it is difficult or impossible to visit a dentist.

**Other Considerations**

The quality of dental care is improving but the utilization of dental services is reducing.

The oral health of those aged 16 years or under seems to be improving along with the involvement of school teachers who provide instruction in oral hygiene [13].

Higher family income would improve the situation as it would increase the utilization of the dental services (more frequent visits to dentists) [11].

#### Croatia

**Epidemiology**

There has never been a national survey of caries prevalence in Croatia. The most recent survey was in 2013 in south-west region of Croatia [14]. The results of this study of nearly 2,000 children aged 6 years who lived in Primorsko-Goranska Zupanija indicted a mean dmft of 4.6.

The last survey of 12-year-old children was in Zagreb in 2010 and the mean DMFT was 5.9 [15].

There is information on the number of children who received orthodontic treatment in 2013, funded by the Croatian Health Insurance Fund:

- Treatment with a fixed appliance 5,802 children
- Treatment with a removable appliance in one jaw only 6,165 children
- Treatment with removable appliances in both jaws 1,195 children

**Prevention**

There was a national prevention program, but it stopped 8 years ago. Today, the Croatian Health Insurance Fund together with Croatian Dental Chamber organizes two educational programs (Super Six and Healthy Teeth go to School).

In the last 2 years, a health education program has been implemented in elementary and secondary schools (10 h during the year). It includes a 1 h lecture on how to maintain good oral hygiene and is given in the 1^st^ grade.

In Zagreb, the capital city sometimes oral hygiene education is provided in schools and kindergartens.

The most important aspect is that when parents enrol their children at school, they have to bring the certificate from a dentist that the child has all teeth “repaired”.

Most of the prevention programs have been organized in urban centres. They take place mostly in schools or in dental offices (cabinets/clinics).

**Treatment and Payment**

For children and young people under 18 years of age almost all dental treatments are covered by the national health insurance scheme. The treatments that are free to the children and their parents are as follows:

- all extractions and all oral surgery treatments
- amalgam and composite fillings, ART fillings, fissure sealants
- all endodontic treatments
- all periodontal treatments
- metal and acrylic crowns on traumatized teeth
- removable dentures
- orthodontic treatments (open bite >3 mm, cross bite >2 mm, deep bite >6 mm, reverse bite, dental impaction, hypodontia, cleft lip and palate, ankyloses of primary teeth,)
- prevention, education, fluoridation

Dental treatment is provided in state (public) dental offices or private ones that have a contract with state health insurance scheme (93 % of all private dental offices in Croatia have such a contract).

Specialist orthodontists and children’s dentists also have contracts with the Health Insurance Fund (Table 9).

**Dental Personnel**

Only general dentists and some specialists in orthodontics and children’s dentistry provide oral health care in Croatia. There are no dental hygienists or other clinical auxiliaries.

The Croatian Health Insurance Fund requires that the dental team consists of a dentist and dental nurse. As a result, all dental offices with a state insurance contract employ a dental nurse.

The dental nurses only provide chair-side assistance, without the possibility of performing dental treatments.

It is planned to open a school for dental hygienists.

**Uptake of Oral Health Care**

It is not difficult to visit a dentist in all parts of Croatia but there are no data on uptake of services.

**Other Considerations**

The economic situation in the last 6 years has had almost no influence on the provision of oral health care in Croatia. Oral health care has continued to be fully covered for children.

It seems that the oral health of the children aged up to 16 years has remained the same in the last 20 years, without significant change, due to the free public health insurance for children.

Judging by the little change in mean DMFT in the last 20 years, the level of oral health could be further improved, not only by increasing clinical services but also by organizing prevention and educational programs in schools and kindergartens. Dentist-nurse teams have to be re-established to work only on child oral health, prevention programs and education.

The analyses of the oral health data for 12-year children in Croatia published by the World Health Organization showed its improvement until 1992 [16]. In 1985 estimated mean national DMFT for 12-year-olds was 6.5 and it dropped to 3.4 in 1990 [17]. In that period every kindergarten was under the control of a children’s dental office and every school had its own dentist.

During the war in former Yugoslavia in the 1990s, abolition of children’s specialist offices for oral health care and stopping the preventive measures in kindergartens and schools adversely affected the oral health status of children. Therefore, by 1999 estimated mean national DMFT for 12 year-olds increased to 4.1. To improve the situation in 1994 parents were allowed to choose the dentist for their child. Unfortunately, because of the lack of knowledge and time, most parents choose their own general dentist. As traditional dental health care includes mostly treatment procedures, whilst individual prevention depends greatly on dentist’s available time and the motivation of the parents to bring the child for check-ups even when no problem exists, this change has had an adverse effect on child oral health in Croatia [18]. Also very few dentists specialize in Children’s and Preventive Dentistry because after training they return to general dental practices and because it is only taught at the Faculty of Dentistry [19].

#### Czech Republic

**Epidemiology**

In the Czech Republic, the most recent national survey of 5-year-olds was in 2009–2010 [20].

The main results were: Caries free 51.8 %, Mean dmft 2.9, SiC 7.19 and Restorative index 21.23 %.

The survey also assessed 12-year-olds and the main results were: Caries free 34.5 %, Mean DMFT 2.14, SiC 5.0 and Restorative index 69.4 % [21].

There are no national or regional data for orthodontic treatment need, oral hygiene and dental trauma in 16-year-olds.

**Prevention**

At a national level, there is a preventive program in primary schools “*Healthy Teeth* for 6–8-year-olds”. There is a regional preventive program in kindergartens “No *more decay* for pre-schoolers”. It is conducted in Prague and its surrounding county. Also there is a regional programme “*Bright smile-Colgate”* in the city of Brno and its surroundings.

**Treatment and Payment**

For children and young people up to 18-years of age, the following treatment are fully covered by the Health Insurance Fund (HIF): check-ups twice a year, local anaesthesia, simple fillings, endodontics, extractions, removable orthodontics. Composite fillings, fixed orthodontics and prosthodontics are partly covered by HIF and up to 20 % must be paid by the patient or their parents. Treatment is provided in private dental offices, public clinics and dental school clinics. Specialist children’s dental care is provided in paediatric sections of dental departments of Medical Faculties (teaching hospitals) and dental departments of some county hospitals

**Dental personnel**

General Dental Practitioners (GDPs) provide general dental care for children. Orthodontists provide orthodontic care. All GDPs are required to work with a chair-side assistant (Dental Nurse), who is trained to provide chair-side clinical assistance, maintain infection control procedures and provide administrative support.

Dental Hygienists are trained and employed. By 2012, just over 600 had graduated, of whom 380 were working in dental offices.

**Uptake of oral health care of children and adolescents**

In 2010, a national survey indicated that the following percentages of children had been seen by a dentist: 0–3 years-old - 66.7 %, 3–6 years-old -66.4 %, 6–10 years-old -85.2 % and 10–14 years-old - 88.7 % [22].

Inequalities in dental attendance were seen in three counties where there was higher unemployment, more people in lower socio-economic groups and poorly motivated care-givers.

**Other considerations**

In some rural areas there is slightly decreasing interest of patients in advanced dental procedures requiring a higher patient financial contribution.

The oral health of children aged up to 16 years is slowly improving [23].

To improve the situation there is a need for:

- An increase in the number of kindergartens and elementary schools involved in school-based preventive programmes and to increase their efficacy.
- An increase in the remittance for the children’s dental care by the GIF to make it more attractive for GDPs.
- The establishment of the official specialty of children’s dentistry.
- A reduction in national inequalities in the provision of special care in children’s dentistry.

#### Former Yugoslav Republic of Macedonia

**Epidemiology -**

The last national cross-sectional study was performed in 2007. Children aged 6, 12 and 15 years were examined.

The national mean DMFT for 12-year -olds was 6.88. The prevalence of orthodontic anomalies in 6-year-olds was 28.17 % and 48.9 % in 12-year-olds. Overall, a CPITN score of 1 or higher in 15-year-olds was present in a mean of five sextants. There are no data on 16-year- olds with traumatized anterior teeth.

**Prevention**

There are national programs for:

Caries prevention through: mechanical and chemical control of dental plaque, application of fluorides, endogenous fluoride prophylaxis by intake of fluoride tablets and fluorinated milk, dietary advice on sugar-intake, fissure sealing, oral health education and motivation.

Prevention of periodontal diseases by monitoring periodontal health by recording CPITN scores.

Prevention of malocclusions through early detection of teeth-jaw discrepancies and referral for orthodontic treatment.

These programs take place in the public sector and are carried out by specialists in children’s dentistry. General dentists, who are included in the preventive program, participate in their offices or in schools [24, 25].

**Treatment and Payment**

For children under 14 years of age, the following dental treatments, which are covered by the national health insurance, are provided free of charge:

Routine oral examinations, providing oral hygiene advice, consultations, plaque control, dental plaque removal and preventive treatments (application of topical fluoride and fissure sealants) and removable orthodontic appliance/prosthesis [26].

All other treatments have to be paid for by patients or others. There is no free service for restorative, endodontic, oral surgery and fixed orthodontics appliance. Free treatment is provided only in public clinics. In private offices (cabinets/clinic) patients have to pay for everything except for plaque control advice.

**Dental Personnel**

There are about 147 preventive teams, which included specialists in children’s dentistry or general dental practitioners and dental nurses and who practice only preventive dentistry.

There are specialists in orthodontics.

General practitioners (as family dentists) and specialists in children’s dentistry who work at University Dental Clinic Centre provide invasive treatment for children. It is obligatory for all dentists to employ dental nurses. Dental nurses’ duties include sterilization of instruments and equipment, preparing restorative materials and for impression taking, cleaning and sterilising instruments.

Dental Hygienists are not employed in Macedonia at present as they are not legally recognized.

**Uptake of Oral Health Care**

There are no data on the percentage of those aged 16 years or less who visited a dentist in the last year. There are no parts of Macedonia where is impossible to visit dentist.

**Other Considerations**

The economic situation has not influenced the provision of oral health care very much. However, some dentists are using dental materials which are not high quality but are cheaper.

There are too many dentists and some are working for nothing.

The oral health of children is improving slightly but not as much as was expected.

There are too many dentists in Macedonia. About 3,000 are registered by the Dental Chamber and about 200 students are entering Dental Schools each year at four Universities. The dentists are not motivated and many cannot charge adequate fees for private dental services.

There are different categories of dentists: public/preventive teams/, concessioners, private dentists who work for the Health Insurance Fund and purely private dentists.

The following measures are needed to improve this situation:

- Oral health care and prevention for children should be provided by all dentists (general practitioners). A restricted number of preventive teams (5–7 teams could be enough) to be supervisors of children’s oral health and to take overall responsibility for planning.
- There is too much attention on fissure sealing in the national strategy (at huge expense without the expected results) [41]. The National strategy should be rationalized (fissure sealants should be based on an individual risk evaluation).
- The evidence for the anti-caries efficacy of milk fluoridation is very limited but fluoride toothpaste is the most widespread and significant form of fluoride used globally and the most rigorously evaluated vehicle for fluoride use.
- Educational forms of prevention are not developed properly. Improving oral hygiene among the children should be the imperative in preventive measures. There should be implementation of oral health promotion programs for preschool and school children.
- The number of students who entering the Dental Faculties should be restricted.
- Better coordination between different categories of dentists is needed.

#### Georgia

**Epidemiology**

The last national survey of oral health in children in Georgia took place in 2012 [27].

First, Seventh and Tenth Grade children (6, 12 and 15-year- olds) were examined.

The caries experience was: dmft 4.57 (SD = 3.42) for 6 year-olds; DMFT 2.04 (SD = 2.02) for 12-year-olds; DMFT 3.51 (SD = 3.14) for 15 year-olds [28].

In the 15 year olds the mean pocket probing depth was found to be 3.34 ± 0.57 mm with a range of 1 to 10 mm and 34.26 % presented with pockets of 5 mm or deeper. Males presented with more plaque, calculus and probing depths than females. When comparing urban and rural population, urban participants presented with more plaque, probing depths and bleeding on probing. Pocket depths were found to be related to the presence of plaque calculus and bleeding on probing [29].

The overall prevalence of Traumatic Dental Injury (TDI) among 12 and 15-year- olds was found to be 10.5 %. The prevalence of TDI was greater in the older age cohort (*p* = 0.0443). Lip posture did not seem to have a marked effect on TDI. Children with an over-jet greater than 5 mm were more likely to present with dental injuries compared to children with an over-jet equal to or smaller than 5 mm (*p* = 0.0411). Children from rural areas presented with greater prevalence of TDI compared to their urban counterparts (*p* = 0.0187) [30].

**Prevention**

There are no state oral health education or prevention programs in Georgia.

Some local programs take place - in schools or in dental offices (cabinet/clinics) [27].

**Treatment and Payment**

There is no free routine oral treatment for children up to the age of 16 years. Only urgent medical services are free.

Children’s Dentists and Orthodontists provide treatment for children.

There are no general dentists in Georgia. Prior to 2014, there were three Children’s Dentistry specialties (Therapeutic Dental Paediatrician, Paediatric Dental Surgeon and Paediatric Maxillofacial Dental Surgeon) and Orthodontics. Since February 2014, as a result of an order from the Minister of Health, Children’s Dentistry is no longer a specialty and children will/can be treated by all dentists and Orthodontists.

Approximately 20–25 % of dentists employ dental nurses (chair-side assistants).

Dental nurses tasks are limited to chair-side assistance only, no dental procedures are carried out by them.

No dental hygienists are trained in Georgia, as there is no such a specialty/subspecialty among the list of medical-dental specialties [27].

**Uptake of Oral Health Care**

There are no official data on the percentage of children aged 16 years or less who visited a dentist in the last 12 months?

In rural areas it is difficult or impossible to visit a dentist. This is because there are no dental clinics or chairs in the village health centres and patients have to travel to the cities in order to obtain dental services.

The current economic situation has had no influence (neither improvement nor decrease) in the last 6 years on the provision of oral health care.

Oral health of children up to age 16 years is not improving, due to the lack of oral hygiene habits, oral health education, oral health prevention programs and high environmental pollution (specifically in the capital Tbilisi) [31].

Educational programs, teaching oral hygiene habits and raising awareness of the importance of oral health should be elaborated. Also preventive programs offering free pit and fissure sealing, fluoridation of drinking water (fluoride content in the drinking water of the capital Tbilisi is only 0.1 mg/l) should be introduced.

#### Greece

**Epidemiology**

The last published oral health survey in Greece was conducted from October 2001 to May 2005. It was a national pathfinder survey (n = 6,048, including adults) [32]. A new survey was conducted in 2013 (results not available yet). Children aged 5, 12 and 15 years were examined. The main results of the 2001–2005 survey are shown in Tables 10 and 11.

**Frequency of toothbrushing**

12-year-olds (self-reported): 31.3 % two or more times a day, 65.3 % sporadically or once a day, 3.4 % never.

15 year-olds (self-reported): 40.4 % two or more times a day, 58.6 % sporadically or once a day, 1 % never.

The Need for Orthodontics was found to be:

- 12 year-olds: 48.7 %, with 13.7 % under treatment.
- 15 year-olds: 42.6 %, with 13 % under treatment.

**Prevention**

There are two programs in Greece to prevent oral diseases in children. They are:

1. An oral health promotion program
2. An oral health month

These programs are both national and local, focusing predominantly on health education and it is unclear what proportion of the population is covered. In addition, there are school dental visits, and oral health promotion activities through the media (TV and magazines) [33].

**Treatment and Payment**

There is free dental care for some types of treatment. More specifically, at state-funded health centres which predominantly cover semi-urban and rural populations while urban populations can be covered through the largest national insurance fund. All this is now in a transition period as a result of a reform of the health sector. The situation may be different in the near future. In terms of the specific types of dental care covered, there are:

- State-funded health centres (n = 202) which provide treatment for rural populations up to the age of 18 years. This treatment consists of: annual consultation, prevention (dietary and oral hygiene advice), topical fluoride applications, fissure sealants, radiography, and restorative treatment. No prosthetic treatment is provided.
- A large national insurance fund (the “IKA”) provides free treatment for urban populations up to the age of 18 years, which includes orthodontics and restorative care but no prosthetic treatment [34].

For adults free treatment is available in:

- Public Clinics
- Some funds contract with private dentists who are remunerated on a fee-for-service basis according to a predetermined tariff.
- Other funds offer their beneficiaries a free choice of dentist. Patients pay the dentist who is then reimbursed by the fund.
- Preventive dental services are not covered [34].

All this is in process of changing.

**Dental Personnel**

Oral health care for children is provided by general dental practitioners and orthodontists

A variety of children’s dentistry “specialists” exist although they ate not officially recognised. Only oral surgery and orthodontics are officially recognized as specialties.

The vast majority of dentists who work either privately or in the public sector do so without dental nurses. Despite Greece having the highest dentist to-population ratio of the European countries, it has very few dental nurses/dental assistants and the exact number of dental nurses is unknown.

There are no dental hygienists in Greece. It is difficult to justify why this is the case.

**Uptake of Oral Health Care**

In teenagers aged between 15 and 18 years, only 7.3 % had not visited their dentist in the past 12 months, 51.6 % had seen their dentists on one or two occasions whereas 41.1 % had seen their dentist on three or more occasions [34].

Apart from in some remote islands/villages, in the mountains, there is no difficulty in finding a dentist.

**Other Considerations**

There is literature in the health services research and public health areas on the influences of the economic crisis, but not specifically on oral health.. The key findings are:

- Adverse social consequences (homelessness, surging crime, people into care).
- Budget cuts (around 40 % of hospital budgets).
- Increased admissions to public hospitals (private health insurance is no longer affordable for many Greeks).
- A 40 % rise in suicides and self-rated general health has deteriorated.
- “Interaction of fiscal austerity with economic shocks and weak social protection has escalated the health and social crisis [35].

It is difficult to say whether or not the oral health of Greek children is improving or deteriorating. The results from the most recent survey will provide more information. Comparisons cannot be made with data from the previous pathfinder studies.

**What needs doing to improve this situation?**

There are potentially many options available for further improvement, particularly if one considers the very limited public health policies that are currently in place. Indicatively, some suggestions could be:

- Health promotion (not just health education) initiatives that consider the common risk factor approach and include oral health and its determinants within a broader health focus
- Public health action with emphasis on the broader social determinants of health
- Targeting vulnerable and hard to reach groups for provision of necessary package of care.

#### Hungary

**Epidemiology**

The last national study was organized in 2008 – but the data are mostly unpublished, most of them are available only through personal communication.

The age group 6-year-olds and 12-year-olds were examined.

Main results of the last study were as follows:

*6 -year- olds:*

Mean dmft: 3.26 (dt:2.94; mt: 0.07; ft: 0.25) [36].

Caries free children: 41.1 % [36].

*12-year-olds:*

Mean DMFT: 2.4 (DT: 1.2; MT: 0.09; FT: 1.1) [37].

Caries free children: 41.1 % (the same as for year olds).

The mean CPI scores were: score 0: 38.5 % (boys: 34.8 %; girls 42.5 %), score 1: (bleeding): 27.1 % (boys: 27.6 %; girls: 26.5 %), score 2: (bleeding and calculus) 34.3 % (boys: 37.7 %; girls: 30.7 %) [36].

**Prevention**

There are some programs to improve oral health of children (and adults also) but these are often “one-off events.”

Currently there is no government/national or regional preventive program in Hungary. Local programs could be organized by any persons/organizations but usually these are only short (1-day) programs with instruction and motivation of the invited or visited persons e.g, dental students to organize preventive programs in different kindergartens or elementary schools but not on a regular basis.

They take place in schools or at other places but usually not in dental offices.

**Treatment and Payment**

Oral treatment, emergency care, examination, and diagnosis, conservative treatments including fillings and endodontics, prosthetic and periodontal treatments, extraction are free for children up to age of 18 years in public clinics and in private dental offices that have a contract with the National Health Insurance (NHI). However, for crowns and bridges, implants, and any other prosthetic or orthodontic appliances, patients have to pay a co-payment for the technical costs (e.g. in case of removable orthodontic appliance 15 % of the cost is paid by the patient and 85 % by the NHI). The whole technical (laboratory) fee for an appliance is unlimited and it depends on the dental laboratory concerned.

**Dental Personnel**

Oral health care for children is provided by general dentists and specialists in children’s dentistry. Orthodontists usually do not contract with the NHI and work on a private basis.

All dentists employ dental nurses as chair-side helpers as this is compulsory in Hungary.

The tasks of dental nurses are to ensure that the equipment is sterile (changing the used dental equipment after every patient) and that the necessary instruments are on the bracket table of the dental chair. Nurses mix the dental materials and give them to the dentists and help with patient record keeping and administration.

Although in Hungary there is an organized system for education of dental hygienists there are not many. Many are not working as dental hygienists because they are perceived as not being cost effective. Most of the private dentists perform those treatments which would be in the competence of a dental hygienist and the fees are included in their fees. In the last 6 years the number of patients who are treated in private dental offices has reduced, so most of the private dentists don’t employ and pay dental hygienists. In other dental offices with an NHI contract most of dental hygienists are working as dental nurses. They may perform some interventions but without extra pay.

**Uptake of Oral Health Care**

There are no data but, if the school dentistry system is working properly (and officially it should be), every child visits a dental clinic at least once a year.

There is no part of Hungary where would be impossible to visit a dentist but there are some areas where the dentist can only be reached by public or private transport and this can cause difficulties. The main reason for this situation is the geographical distribution of dentists – there are some areas in Hungary (mainly in the eastern part of the country), which are not so popular because these areas have more socio-economic problems (e.g. many more unemployed people) than Budapest.

**Other Considerations**

As a result of the financial crisis during the past six years the situation of general and oral health care has become worse. Prevention should be very important but it would need a lot of money especially in the first period of a preventive program. The Government is unwilling to spend money on prevention when the results are only apparent after 4 years or more (the usual term for which a government is elected).

According to the national data, overall, the oral health of children seems to be improving slightly but this is only on average and includes data from different areas of the country. However, the socio-economic circumstances are different so dental health data from all parts of the country can show great differences. In addition, the data indicate that as far as the prevalence of caries free 6 year-olds is concerned, there is a great difference not only between the different regions but between urban and rural areas. It may well be that this slight improvement which is shown by the national data does not represent the real situation even in a small country like Hungary. Also there are comparable data only for 6 and 12-year-old children, and data for children of other ages are unknown.

It is necessary to publish data and influencing factors in different areas for children of ages other than just 6 and 12 years. Then the data and descriptions of factors influencing oral health could provide basic information for planning a successful national program with regional specialties in the dental care system. Then the Government could be asked to fund a preventive program prepared and organized by relevant and experienced persons who understand the situation.

#### Latvia

Health systems in the Baltic States, including Latvia, are structured according to a typical Eastern European Model [1, 2]. However, there has been a shift from predominantly public service provision to private service (only 11 % of oral health care is now provided within the public service). Oral health insurance schemes are still developing. There is limited, but growing provision of free treatment for under 18 year olds, a growing number of dental hygienists, dental nurses and assistants (ratio dental personnel: dentists 57 %: 43 %), improving oral health for children and young people (a decline in caries decline by 40 % over the last 20 years), a growing rate of regular attendance for oral health care (50–60 % of the population annually) and mainly women working in dentistry (89 % as dentists and 98 % as dental hygienists). Care for adults is privately financed. As mentioned above, since 1995, there has been a 40 % decline in dental caries in children and young people [38], but not for adults.

**Epidemiology**

In 1995, in recognition of high caries levels in all age groups, a National Preventive Program in Dentistry was initiated by the Oral Health Centre (Institute of Stomatology), in close cooperation with the State Dental Centre. The program was divided into five blocks and the responsibility for the program was taken by both the education and health care systems. There were two phases (1995–2004 and 2005–2010). During that period 26 local Oral Health Centres were created.

Assessment and effectiveness for preventing and curative work is based on regular collection of oral health data in defined age groups. The last national oral health study was carried out in 2011 by the Centre of Dentistry and Facial Surgery. The study was conducted according to the WHO (1997) protocol for diagnosing caries, sample selection was randomized and the sample size provided a confidence level of 95 %. The study population comprised 5, 448 12-year- old children. The mean DMFT was 3.35, 21.3 % were caries free. Frequency of tooth brushing was: once a day - 33,1 %, twice a day – 51.5 % [38–40].

For 6-year-olds the study population comprised 4, 297 children. The mean DMFT was 0.21, and dmft 4.84 [40].

Every year there are reports about the oral health situation published in the Statistical Yearbook of Health Care in Latvia 2012. For the 200 282 children, who had received dental care services, mean DMFT for 6-year- old was 0,6 (in 2011) and 0,7 (in 2012), DMFT for the 12-year-olds was 2.9 (in 2011) and 3.0 (in 2012) [41].

**Prevention**

Prevention in Latvia is based on: the principles of health promotion, a common risk factor approach, developing a whole population strategy and involving dental and general health teams. School councils, the National Health Agency, media and industry are also all involved.

During the period 1995–2009 there were two kindergarten and three school based oral health education programs, covering about 150, 000 children every year (55 % of the population aged 0–17 years). The programs were introduced in partnership with the dental industry, the Oral Health Foundation and local Oral Health Centres.

The Baltic kindergarten program “Tooth and Friends” (Wrigley) was operational from 2001 to 2011, every year it covered 22, 000 children. With the economic crisis the programs were closed.

A Puppet “Smart Tooth”- road show together with the mobile dental clinics covered about 10, 000 children in the years 2009–2011. The Centre of Dentistry and Facial Surgery. kindergarten program “Tooth and Friends” had a target group of 5–7 years of age (the oldest age group in kindergartens). Topics covered were: healthy breakfast, cleaning teeth after breakfast, proper toothpaste and brush usage, a proper “diet clock”, sugar free gum after meals and snacks, the significance of saliva in oral healthcare, teeth and cavity monsters - “think before you eat sweets”, How children’s teeth change, visiting the dental hygienist and dentist twice a year and cleaning teeth before bed time.

**Oral health promotion and education programmes**

A supervised tooth brushing program still covers about 65 % of the kindergartens in Latvia in cooperation with the local Oral Health Centres (OHC). A nursing bottle caries campaign ran between 2012 and 2014 and was organised by the Centre of Dentistry and Facial Surgery (CDFS). Future plans include a new program for kindergarten teachers, parents and children. It will involve the Oral Health Foundation (OHF) in cooperation with the Riga city council.

Next year there will be a program for pregnant women, new mothers and their children - (OHF) in cooperation with the Centre for Disease Prevention and Control (CDPC) and a dental trauma prevention program as part of trauma campaign and a program for preventing smoking by young people.

**Treatment and Payment**

Oral health care for children up to the age of 18 years is publicly financed (with exception of orthodontic treatment) from the Sickness Funds (since 2012 by the Public Health Agency).

Oral health care is provided by: general dentists and specialists in children’s dentistry (2 % of dental personnel), who have agreements with the Public Health Agency. orthodontists (1 % of dental personnel). Only orthodontic consultations are free. However, children with severe orthognathic anomalies can receive all orthodontic treatment free until the age of 22 years.

**Dental Personnel**

There is one dental faculty located in the Riga Stradiņš University. Each year its entry consists of 50 dentists and 25 dental hygienists. Dental hygienists’ education commenced in 1995, and since then 295 dental hygienists have graduated. The ratio of dental hygienists : dentists is now 1: 6), 24 % of Dental Hygienists are working in Public Health. Other dental personnel are educated at Riga’s First Medical College. Dental care professionals are obliged by legislation to maintain and develop their professional skills – mainly through continuing education delivered by the professional associations.

In Latvia (in 2012) there were 1474 dentists who constituted 40 % of dental personnel.

The other dental personnel were:

- Dental therapists 87 - 2 %.
- Dental hygienists 219 - 6 %.
- Dental nurses 1061 - 29 %.
- Dental assistants 299 - 8 %.
- Dental technicians 551 - 15 % [36, 37].
- All dentists have dental assistants or dental nurses in their dental practices. Dental nurses are working as chair-side dental assistants and also doing preventive work with patients.

**Uptake of Oral Health Care**

In 2012, the average cost of oral health care per child was 39.31 EUR per year and covered approximately 53 % of all children up to the age 18 in Latvia [40, 42]. The reasons for visiting dentist were: regular check-ups 83.4 %, dental trauma 0.2 %, not mentioned 2.8 %, acute pain 13.6 % [40].

As Latvia is a small, but densely populated country (total population just over 2,000,000), there were problems with access to oral healthcare. They were solved in 2007 by introducing mobile dental clinics. There are two such clinics dental clinics, the number of visits per year to these mobile clinics has increased from 3,457 (in 2008) to 8,402 (in 2012) [36]. The mobile dental clinics provide dental care for children and adults in rural districts and for physically disabled and institutionalized persons in both urban and rural areas.

**Other Considerations**

The economic situation in the last six years has not directly influenced the financing of the Public Health Agency - regarding the total expenses of dental care of children. However, the budget for the health care in Latvia has not yet returned to the level of 2008. In addition, the socio – economic situation has influenced the population’s oral health behaviour and tooth brushing habits became worse. There is increasing number of children treated under general anaesthetic at the Institute of Stomatology (from 180 cases in 2006 to 926 cases in 2012) [43]. Mean DMFT is not declining, it is remaining at the same level in all age groups. Prevention must be based on: the principles of health promotion, a common risk factor approach, developing a whole population strategy, involving dental and general health teams, school councils, National Health Agency, media and industry.

In 2011, new Public Health Strategies in Latvia (2011–2017) were developed at the Ministry of Health. Key strategic directions for action are to: reduce health inequities, ensure high quality and assessable care services for all inhabitants, health promotion and disease prevention. The Centre for Disease Prevention and Control (CDPC) was established in 2012 with the mission to implement public health policy on epidemiological surveys, patient safety, disease prevention and health promotion. New guidelines were formulated at the Centre of Dentistry and Facial Surgery. They relate to a management system for good dental care and patient safety in oral health care. The competent authorities maintain dental staff registration and dental practice accreditation is the National Health Agency and Health Inspection. A Baltic Oral Health Strategy is currently being developed by the Latvian Dental Association in cooperation with Estonian Dental Association and Lithuanian Dental Chamber.

#### Lithuania

**Epidemiology**

The last national survey was in 2013 and 4–6 year-old children were examined. The mean dmft was 7.9 and the mean DMFT was 1.12. Caries prevalence was 89.7 %. A previous national survey of 7–12-year-old children took place in 2007–2008. In this survey, for 12-year-olds, the mean DMFT was 2.0 [44].

**Prevention**

There is a national fissure sealant program. It takes place in private and public dental offices.

**Treatment and Payment**

Other than orthodontics, which is partly free, other treatment for children and young people aged 16 years or less is free. Orthodontics for aesthetic, rather than functional reasons, has to be fully paid for by the child/young person’s parents.

Oral healthcare for children/young people is provided in clinics owned by the Lithuanian National Health System (LNHS) or in private dental offices that have a contract with the LNHS for reimbursement from the Compulsory Health Insurance Fund.

**Dental Personnel**

General dentists and specialists in children’s dentistry (58 in Lithuania) provide treatment for those who are 16 years of age or less. About 50 % of dentists employ dental nurses. Dental nurses’ tasks are to prepare and maintain the clinical environment, chart during oral examinations, prepare and mix materials.

**Uptake of Oral Health Care**

There are no data on the percentage of those aged 16 years or less who visited a dentist last year. Access to oral health care can be difficult in areas of low population density. This is because some rural areas are far from public dental clinics and there are no private ones. Public transport to such areas is often very poor.

**Other Considerations**

There are no data on how the economic situation in the last 6 years has influenced the provision of oral health care in Lithuania. However, anecdotally, the oral health of those aged 16 years or less has changed little in the last 6 years. Nevertheless, as can be seen from the 2013 survey of 4–6 year- olds, the prevalence of dental caries is very high. Financial resources and political commitment are required to improve this situation.

#### Moldava

**Epidemiology**

The oral health condition of children from the Republic of Moldova was assessed in 2010–2012 as a result of national study of the oral health which was performed according to the WHO (1997) criteria. It involved the clinical examination of 2,461 children who were 6, 12 and 15 years old and who came from urban and rural areas. The study results indicated that the mean national DMFT for 6 -year old children was 0.28 ± 0.21, in 12- year old children - 3.64 ± 0.26 and in 15 -year old children - 4.32 ± 0.31. The study found that 85.45 % of children from rural areas did not perform good oral hygiene and rarely were given new tooth brushes. There was also little motivation to create a healthy environment. The average of OHI-S index for children from rural areas was 1.73 ± 0.36. Whereas the OHI-S index from urban areas was 0.82 ± 0.24.

It is an alarming fact that in most regions of the country, especially in rural areas, the number of children with untreated carious teeth, oral or general complications, or teeth extracted as a result of early dental caries has been constantly increasing. This tendency is found at all stages of oral development (primary, mixed and permanent dentitions) demonstrating a high need for care and treatment, an insufficient number of preventive examinations, a low number of visits to the dentist and also a delay in dental treatment until the complications occur. The prevalence of oral abscesses has increased by 5.2 %, spreading infections of oral origin by 3.6 %, cases of septicaemia by 4 %, and deaths due to complications of oral infections by 2 %. There is a constant increase in the number of gastrointestinal, cardiovascular, excretory, etc. disorders, which appear mainly because of persistent foci of chronic odontogenic infections [45]. At a national level the need for orthodontic treatment and frequency of trauma to the anterior teeth has not been studied.

**Prevention**

In order to improve their oral health a National Oral Health Program for children in Moldova was developed during the period of 1998–2007. However, due to lack of funding, the program has only partly taken place in Chisinau. Nevertheless, the prevalence and incidence of major dental diseases, dental caries and periodontal diseases were reduced in children from Chisinau.

Recently the National Oral Health Program has been developed and is planned for implementation during 2014–2020. At present the preventive measures are implemented at national and local level, and take place in dental offices, schools or public clinics.

**Treatment and Payment**

The treatment of some oral disorders is free for children up to 16 years and is performed according to the unique program of compulsory health insurance, which includes the emergency dental care and one preventive consultation per year for children aged up to 18 years and pregnant women. It is provided only in public clinics. Free emergency dental care for children is provided in the following cases : acute pulpitis, acute apical periodontitis, exacerbating apical periodontitis, acute aphthous stomatitis, acute necrotizing gingivitis and stomatitis, abscesses, pericoronitis, post extraction complications, oral bleeding, acute lymphadenitis, acute osteomyelitis, acute and calculus related sialadenitis, acute and odontogenic sinusitis, trauma to and fractures of the jaw and tooth extractions. Preventive consultations for children under the age of 18 years and pregnant women include examination of the oral cavity and oral hygiene advice.

**Dental Personnel**

Oral Health Care for children is provided by general dentists, specialists in children’s dentistry and orthodontists. About 20 % of dentists, mostly from private clinics, employ dental nurses. They assist the dentist in all aspects of patient care, which will includes getting the appropriate instruments ready, mixing materials and ensuring patient comfort, taking notes from dentist’s dictation for records and sterilisation of all the instruments.

There are no trained dental hygienists in the country, since, at present there is no such specialty as “dental hygienist “included in the legal statutes for healthcare specialists.

**Uptake of Oral Health Care**

About 68 % of children up to 16 years of age visited the dentist during the last 12 months. For children from some rural areas, it is difficult and sometimes even impossible to visit the dentist because of the lack of specialists and dental offices in these localities. There is limited dental care access for some socially vulnerable categories of the population due to inability to pay the transport (to the dental clinics) and the cost of treatment which is not insured by state. It is unquestionably important to improve the lack of motivation for this category of population in maintaining and improving oral health.

**Other Considerations**

The price of dental services in the Republic of Moldova is the lowest in Europe. However, it is still unaffordable for 35–40 % of the population. At present, the poor state of dental health care for children is alarming. The private sector which has appeared and developed over the past two decades, provides high quality, but very expensive dental services which are inaccessible to most people. In this regard, many children and adolescents do not seek dental care unless it is an emergency and they ignore non-painful dental problems. The most vulnerable category of the population is children who live in rural areas.

The treatment of oral diseases requires considerable financial resources that cannot be covered by the National Health Insurance of the Republic of Moldova. At present, the annual funds for oral treatment and preventive measures for example for a 1-year old child is totally insufficient (about 47 lei per year, that is 2.81 EUR).

At present, the oral health condition of children aged up to 16 years from the Republic of Moldova is deteriorating.

The high degree of morbidity from oral diseases and also the neglect of preventive measures proves the need to direct oral care to children in order to prevent diseases and to initiate and implement oral health programs in public institutions, which will help to reduce morbidity and improve quality of children’s lives.

Insufficient oral care for children, the lack of correspondence between existing oral care and the actual needs of population, the expensive treatment and insufficient finances, determine the need for significant changes in the healthcare system and social protection of children in the Republic of Moldova. Under the current circumstances the improvement of the oral health condition of children from the Republic of Moldova should be improved as soon as possible by insuring treatment of oral diseases and providing appropriate care and treatment. It is necessary to involve the government, to implement policies, strategies and health, educational, environmental, social or correlated programs over a long period of time, in order to alter the population’s mind set. One of the opportunities to reform children’s oral care is the implementation of National Oral Health Program which is planned for the period of 2014–2020 and compiled according to the strategy of “Healthcare 2020” and the Platform for Better Oral Health in Europe. This program aims to improve the oral health and life quality by implementing effective preventive measures for dental diseases in about 700,000 children from 2,800 educational institutions and schools.

#### Romania

**Epidemiology**

In 1992, the national mean DMFT for 12 years olds was 4.0 [46].

The last national study of children’s oral health in Romania was performed in 2000.

The national mean DMFT for 12 year olds was 2.8 [47].

In the past few years, several regional and local studies have taken place.

**Prevention**

There has been no national prevention program for oral health. However, at a local level a fluoride mouth rinsing program has taken place in schools in Constanta and Iasi counties.

This program was funded by the Ministry of Health, in order to reduce the prevalence of caries in the school children in grades 1–4 (6–11 years old). The program provided oral health education and also introduced supervised fluoride mouth rinsing with a neutral fluoride solution containing 0.275 % sodium fluoride (Fluorostom, produced by the National Institute of Chemical–Pharmaceutical Research, ICCF, Bucharest) every week in grades 1–4 in both counties.

**Treatment and Payment**

Oral treatment was funded by the public health insurance system (PHIS) starting from 1998 until April 2013. Annual check-ups and free treatment were available to children under the age of 18 years. The total amount paid to each dentist, by the PHIS for all patients (approximately 200 euro/month), was very low. For example it only covered the cost two orthodontic appliances [48].

In April 2013, the PHIS stopped funding any dental treatment for all children up to the age of 16 years. However, funding for this age group was resumed in 2014 but at a low level. At present, the vast majority of oral health care and treatment for patients of all ages is provided at private dental offices (clinics), which are known as cabinets in Romania. It has to be paid for by the patients themselves, or for children by their parents.

**Dental Personnel**

General dentists and also specialists in children’s dentistry and orthodontists are provide oral care treatment for children.

The percentage of dentists who employ dental nurses (chair-side assistants) to help them is under 20 %.

The dental nurses perform the following tasks: managing patients’ appointments, making sure all equipment is sterilized and ready before procedures and treatments, operating suction devices during treatment, preparing materials, performing stock control, of dental materials and helping reassure patients.

Dental hygienists’ education- started in 1975 at five state universities, but was stopped after few years. It started again between 1995 and 2002 and restarted in 2007. Dental hygienists are registered by the Romanian Order of Medical Assistants and Midwives. Dental hygienists are currently trained only in Timisoara.

An educational program for dental nurses has existed for some 15 years. It comprises, 3 years of full­time training in colleges, organized by the Dental Medicine Faculties. They pass a qualifying examination and get a license for free practice. They are registered by the Romanian Order of Medical Assistants.

**Uptake of Oral Health Care**

The percentage of the population of several countries in EU Member States in Eastern Europe, who have visited a dentist in the previous 12 months, appears to be low. According to the Eurobarometer study, in 2010 in Romania, it was 34 %. However, as children no longer receive free treatment it seems likely that by 2014 this percentage had fallen. The percentage of children aged 16 years or less that visited a dentist in the last 12 months is very difficult to estimate since there is no national program for data collection.

The parts of our country where it is difficult or impossible to visit a dentist are mainly the rural areas where no dental services are provided. The main problem still remains the lack of money and the fact that the public services do not provide any money for treating these children and thus it remains the most important issue.

**Other Considerations**

The economic situation in the last 6 years has had a strong adverse influence on the provision of oral health care in Romania. As explained previously, in April 2013 funding from National Health Insurance was stopped for 1 year.

There is anecdotal evidence that the oral health of Romanian children aged up to 16 years is definitely deteriorating. Because there is no national program, which could actually monitor the oral status of children, it is impossible to quantify the extent of this problem.

In order to improve this situation, funding (which is obviously lacking at present) is needed together with a national policy, overseen by a competent team, to ensure that it is implemented and maintained.

#### Russia

**Epidemiology -**

The last national study of the oral health of children in Russia was performed in 2008. Children aged 6, 12 and 15 years were examined.

The mean DMFT figures for 6, 12 and 15 year-olds were 0.23, 2.51 and 3.81 respectively. The prevalence of periodontitis (a CPITN score of 3 or 4) among 12 and 15 year-olds was 34 % and 41 % and the need for orthodontics was 55 % and 57 % respectively. The national study was performed according to the WHO 1997 recommendations. It did not investigate oral cleanliness and the number of 16-year-olds with traumatized anterior teeth.

**Prevention**

There are local programs for the prevention of oral diseases among children in Russia. They are within the framework of scientific research and are carried out in preschool institutions, schools, and in dental offices (cabinets/clinics).

A Federal National program the for the prevention of oral diseases was developed by a working group and approved by the Russian Dental Association, but was not implemented because it did not obtain national financial support.

**Treatment and Payment**

All Russian children up to the age of 16 years can receive free oral treatment for surgical, therapeutic and preventive dentistry (extractions, fillings, scaling and polishing) and some items of orthodontics (removable appliances) and prosthodontic dentistry (removable prostheses). Treatment with fixed orthodontic appliances, crowns and fixed prostheses must be paid by the child’s parents or others.

Free dental treatment is provided in public dental clinics, school dental clinics (cabinets, offices), and dental departments and clinics in children’s hospitals but not in private dental offices (cabinets/clinics).

**Dental Personnel**

General dentists, specialists in children’s dentistry and orthodontists provide oral health care for children in Russia.

As a rule, dentists employ dental nurses to help them in every dental clinic but only in private offices/cabinet/clinics do they work as chair-side assistants.

Dental nurses prepare the workplace, instruments and materials ready for a dental appointment. They also perform infection control procedures and help dentists to complete medical and dental documents. In addition, chair-side assistants perform four handed dentistry, assess patient’s oral hygiene level, the teeth and periodontium and give oral health education and preventive measures under dentist’s supervision.

Dental Hygienists are trained and employed in Russia.

**Uptake of Oral Health Care**

There are no data on percentage of those aged 16 years or younger who visited a dentist in the last 12 months.

Some rural populations have difficulties in accessing dental treatment due to a lack of dentists in some villages and their remoteness from dental clinics.

**Other Considerations**

In the last six years in Russia, public funding for dentistry has been reduced, which has led to an increase in paid dental services and costs for patients.

The oral health of children in Russia is improving. In the last 10 years the DMFT figures and prevalence of dental caries and periodontal diseases in children have decreased. However a high prevalence of dento-facial anomalies is present in the population, highlighting the necessity of their early diagnosis and treatment. For further oral health improvement it is necessary to develop and implement preventive programs for urban and rural populations in different regions of Russia.

#### Slovakia

**Epidemiology**

The last oral epidemiologic survey was carried out in 1999, with a limited sample size. The sample size for children was influenced by the necessity for parent’s agreement to the examination of their children [49–51].

Children in age groups 5–6 years old and 12 years old were examined.

The main results were that in 5–6 year-olds, the prevalence of caries free children was 13,4 % and for 12 year- olds, the mean DMFT was 4,3. There are no data on 16-year-olds with traumatized anterior teeth.

**Prevention**

In Slovakia there are some local oral health education programs for kindergartens and primary schools [50]. Generally education about oral health or general health is optional. In dental offices individual programs are offered to patients.

**Treatment and Payment**

There is free oral treatment for children and adolescents up to 18 years of age for periodic examinations, periodic check-ups, polishing and scaling twice per year, fluoridation. Fillings and prosthetic treatment are covered, but with some co-payment from the patient’s parents.

The free treatment is provided in both public clinics and private offices.

**Dental Personnel**

The providers are general dentists and specialists in children’s dentistry and orthodontists.

100 % of dentists employ dental nurses. It is a legal requirement in Slovakia for all dentists to employ at least one nurse/dental assistant. Dental nurses assist dentists, with dental recording, disinfection and sterilization, patient appointment making and scheduling.

Dental hygienists are trained and employed in Slovakia.

**Uptake of Oral Health Care**

There are no freely available data on the percentage of children aged 16 years or younger who visited a dentist in the last 12 months.

In some parts of Slovakia there are difficulties for the population to visit a dentist. This is because of the absence of dentists in villages and small towns and an absence of basic knowledge about the need for oral health in some groups of the population.

**Other Considerations**

In the opinion of the author, oral health in Slovakia is deteriorating. To improve this situation there is a need for:

- Increased attention to primary prevention in the population.
- The establishment of a National Oral Health care program.
- Increased responsibility of parents for oral health, for regular periodic check- ups and for primary preventive methods.

#### Turkey

**Epidemiology**

A cross-sectional study was undertaken in 2013. A total of 3,171 toddlers were included (52 % males and 48 % females). Their mean age was 25.8 ± 10.1 months. The prevalence of Early Childhood Caries (ECC) was 17.3 %, while the mean df(t) was 0.63 ± 1.79. ECC increased significantly with age. Dental caries was mostly observed in primary maxillary central teeth. Occlusal and buccal surfaces were the most affected sites. The difference in distribution of caries between maxilla and mandible was found to be statistically significant (*P* <0.05) [52].

Previously, a cross-sectional study was undertaken between September 2004 and February 2005. The ages/age groups were 5, 12, 15, 35–44 and 65–74 years. At the end of the study, 7,833 individuals had been examined. Only 30.2 % of the 5-year-old group was caries-free, and the mean dmft was 3.7. Mean DMFT was 1.9 in 12- year-olds and 2.3 in 15-year-olds [53]. The mean df(t) was 0.63 ± 1.79. ECC increased significantly with age [52].

In another study, when the dental health component of The Index of Orthodontic Treatment Need (IOTN) was considered, 38.8 % of Turkish school population showed great need treatment, 24.0 % moderate need treatment and 37.2 % slight or no need. On the other hand, the referred population had a 83.2 % great need treatment, 12.0 % moderate need treatment and 4.8 % no need treatment according to the Dental Health Component (DHC) of the Index of Treatment Need (IOTN) [54].

In yet another recent study, the sample consisted of 836 school children (384 male and 452 female, aged 11–14 years). Treatment Priority Index (TPI), scores showed that 36.4 % had normal occlusion, while 41.2 % had slight, 15.7 % had definite, 4 % had severe, and 2.7 % had very severe malocclusion [55].

As far as trauma to anterior teeth is concerned, In a local study in Istanbul, a total of 154 patients, aged 1-13 years, presented with a total of 337 traumatized teeth (255 permanent and 82 primary). Ninety four boys (61 %) and 60 girls (39 %) with a mean age of 7.91 ± 3.15 years participated in the study. Dental injuries were frequent in the 6–12 year age group. The most common type of dental injuries recorded was luxation injuries (43.3 %), uncomplicated crown fractures (20.5 %), and complicated crown fractures (19.4 %). The main causes were falls (55.2 %) and being struck by an object (22.1 %) [56].

**Prevention**

The Toothfriendly Foundation’s caries prevention program was launched in Turkey in 2002. The program aimed at making oral health behaviour a part of daily routine for thousands of school children. In 2010, some Turkish University Dental Institutes took over large parts of the fluoridation program. Last year, the program reached over 17,800 children, of whom 870 were directly treated by the Turkish Toothfriendly team in the city of Istanbul. The Foundation also supplied free toothbrushes and fluoride toothpaste via its partner company and provided the volunteer dentists and teachers with educational material.

The Alliance for a Cavity-Free Future (ACFF) is a worldwide group of experts who are working to stop the initiation and progression of dental caries. Their goal is to establish a Cavity-Free Future for individuals of all ages. In 2013, worldwide leaders in dentistry and public health gathered at the FDI World Dental Congress in Istanbul to kick-off the Turkey chapter of the Alliance. Turkish chapter of the Alliance is joining the global movement towards a cavity-free future in order to build upon the work already being done on the issue of caries by dental and public health professionals in Turkey.

There have been some local studies of preventive dental programmes. A study among 149 children with high-risk group of 11- to 13-year-olds with low caries activity was performed to compare the caries preventive effects of 2-year application of school-based chlorhexidine varnish, sodium fluoride gel, and dental health education programs. There was no significant difference among the groups in the caries increment after 2 years, with mean DMFS +/- SD 0.95 +/- 1.33, 0.88 +/- 1.47, and 1.05 +/- 2.01 in the chlorhexidine varnish, sodium fluoride gel, and education groups. The education group showed a significant increase in the salivary levels of *S mutans* in comparison with the other groups *(P* = .004) [57].

A 4-year longitudinal study to assess the effectiveness of topical fluoride application and a dental health education program was designed to improve the oral hygiene, caries prevalence, decrease the plaque accumulation 640 school children, aged 6–12 years, in Ankara between 2000 and 2003. dfs/DF-S and plaque scores were recorded in all groups. No statistically significant differences were detected between the caries prevalence values of test and control groups and in follow-up examinations, the mean plaque scores were significantly different for test and control groups (*p* >0.05) [58].

The Oral health prevention programs take place in schools and in dental clinics.

**Treatment and Payment**

In Turkey, all types of dentistry are free for the children under 18 years of age. The IOTN index is used to assess orthodontic treatment need. If the children have high enough IOTN scores, the Social Security Institute (SSI) pays all the treatment fees except for laboratory materials and appliances. In addition, orthodontic treatment for patients under 18 years of age can be provided in private clinics and the institution covers the treatment fee within its official prices.

The SSI finances all the free treatment. However, it is only free if provided in public and government clinics and publicly funded universities. In private clinics children’s parents have to pay the fees other than for orthodontics (see answer to the previous question).

**Dental Personnel**

Children’s dentistry is mainly provided by general dentists all over the country in private and public clinics and by children’s dentists in university dental faculties and in some public clinics.

Less than 10 % of dentists employ chair-side nurses who assist and help the dentists.

Dental hygienists are not trained nor employed in Turkey.

**Uptake of Oral Health Care**

In 2012, nearly 35,282,921, (48 %) of the total population of Turkey, visited a dentist.

**Other Considerations**

In the last 6 years, the economic situation may not have directly influenced the financial provision for oral care. There has been no reduction in the amount of public money spent on oral care in Turkey.

It is unclear whether the oral health of Turkish children has improved or deteriorated in the last 6 years.

Tooth brushing programs, a national health program and minimal intervention treatment plans for managing dental caries need to be introduced at home, in family health centres, kindergartens, and schools.

The dental professions, government and universities need to determine the best preventive oral health program for children in Turkey and then implement it.

#### Ukraine

**Epidemiology**

Epidemiologic studies of the oral health of children have not been conducted in Ukraine.

**Prevention**

Advice on the prevention of oral diseases is given in kindergartens and schools.

**Treatment and Payment**

Children’s routine dental care, including check-ups, fillings, preventive advice and extractions, are paid by the public funds (government) if it is delivered in a public clinic.

All treatments are provided free for orphans and disabled children. The treatment is provided in public clinics.

**Dental Personnel**

Oral health care for children is provided by children’s dentists and orthodontists.

Nearly 80–90 % of dentists work with nurses and 20–40 % of dentists work with an assistant dentist. This depends on the region and the ownership of dental institutions.

Dental nurses perform the following tasks:

Participate in the medical - diagnostic process.

Make appointments for dentists.

Assist dentists in dental operations.

Provide emergency first aid.

Clean, sterilise and prepare dental instruments and equipment Prepare impression and restorative materials.

Give oral hygiene advice and prepare patients for anaesthesia.

They can also perform resuscitation techniques and first aid for traumatic bleeding, collapse, poisoning, mechanical asphyxia and allergic conditions.

Help the dentists maintain medical (dental) records.

They are actively involved in the dissemination of medical knowledge among the population on the prevention of dental diseases and their complications.

Dental Hygienists are trained and work with dentists.

**Uptake of Oral Health Care**

All district and regional centres provide the opportunity to visit a dentist, difficulties may arise in small villages where there are no medical/dental facilities.

**Other Considerations**

The economic situation has adversely affected the provision of oral care due to price increases for dental materials and for treatment.

Currently the oral health of children under 16 year-olds is deteriorating.

In the opinion of the author to improve this situation, it is necessary to create a program providing free dental care for the child population.
