# Erratum to: Proceedings of a workshop, held in Constanta, Romania on 22 May 2014, on Oral Health of Children in the Central and Eastern European Countries in the context of the current economic crisis

**DOI:** 10.1186/s12903-016-0269-x

**Published:** 2016-09-15

**Authors:** Dorjan Hysi, Kenneth A. Eaton, George Tsakos, Paula Vassallo, Corneliu Amariei, the DPH Group

**Affiliations:** 1 Albanian Dental Association, University Dental Clinic, Tirana, Albania; 2 European Platform for a Better Oral Health in Europe, Old Saddlers, Canterbury Road, Ashford, London, Kent TN25 4EW UK; 3 European Association for Dental Public Health, London, UK; 4 Council of European Chief Dental Officers, Valletta, Malta; 5 Romanian Association of Oro-Dental Public Health, Constanta, Romania

## Erratum

Unfortunately, the original version of this article [1] contained an error. The Tables and an additional file (cited in the original article as Fig. 1) were accidently omitted.

Tables 1, 2, 3, 4, 5, 6, 7, 8, 9, 10 and 11 can be found below.

**Table 1:** The presenters at the Constanta Workshop Held in May 2014 and authors of the country reports

Country	Name
Albania	Dorjan Hysi
Armenia	Hrant Ter-Pohhosyan*
Belarus	Tamara Tserekhava
Bulgaria	Lydia Katrova
Croatia	Nikola Petricevic
Czech Republic	Zdenek Broukal
Former Yugoslav Republic of Macedonia	Julijana Nikolovska
Georgia	Mariam Margvelashvili
Greece	Antiopi Ntouva** and Georgios Tsakos
Hungary	Melinda Madléna
Latvia	Egita Senakola
Lithuania	Alina Puriene
Moldova	Aurelia Spinei
Romania	Roxana Oancea
Russia	Nadezhda Pazdrikova
Slovakia	Simona Dianiskova
Turkey	Betul Kargul
Ukraine	Kostiantyin Lykhota

* Did not attend in person. His report was presented on his behalf

** Did not attend in person but first-authored report for Greece with Georgios Tsakos as a co-author

**Table 2:** Categories of epidemiological data for the ECC reported by participants in the workshop

Countries	Caries epidemiology surveys			Periodontal status	Oral Hygiene	Orthodontic Treatment needs	Dental Trauma
	6 y old	12 y old	15 y old				
Albania	Yes	Yes	No	No	Yes*	No	No
Armenia	No	No	No	No	No	No	No
Belarus	Yes*	Yes*	Yes*	No	Yes	No	No
Bulgaria	Yes*	Yes*	Yes*	Yes	Yes	Yes	No
Croatia	Yes**	Yes**	No	No	No	No	No
Czech Republic	Yes	Yes	No	No	No	No	No
Georgia	Yes	Yes	Yes	Yes	No	No	Yes
Greece	Yes*	Yes*	Yes*	No	No	Yes	No
Hungary	Yes	Yes	No	Yes	No	No	No
Latvia	Yes	Yes	No	No	No	No	No
Lithuania	Yes	Yes	No	No	No	No	No
Macedonia	No	Yes	No	Yes	No	Yes	No
Moldova	Yes	Yes	Yes	No	Yes	No	No
Rumania	No	Yes	No	No	No	No	No
Russia	Yes	Yes	Yes	Yes	No	No	No
Slovakia	Yes	Yes	No	No	No	No	No
Turkey	Yes	Yes	Yes	No	No	Yes	Yes*
Ukraine	No	No	No	No	No	No	No

*the data are presented in the tables following the country data

**the data presented are from local surveys

**Table 3:** Resume of data on preventive programmes and free treatment for 0-16 year-olds in the ECC reported by participants in the workshop

Countries	Preventive Program		Free treatment service for the age 0-16 years old			
	National	Local	Prophylaxis, restorative treatment	Prosthetic, orthodontic treatment	Public service	Private service
Albania	No	Yes	Yes	No	Yes	No
Armenia	Yes	No	Yes *	Yes *	Yes	Yes
Belarus	Yes	No	Yes	No	Yes	No
Bulgaria	Yes	No	Yes	No	Yes	Yes
Croatia	No	Yes	Yes	Yes	Yes	Yes
Czech Republic	Yes	Yes	Yes	No	Yes	Yes
Georgia	No	Yes	No	No	No	-
Greece	Yes	Yes	Yes	No	Yes	No
Hungary	No	Yes	Yes	No	Yes	Yes
Latvia	Yes	Yes	Yes	No	Yes	Yes
Lithuania	Yes	No	Yes	No	Yes	Yes
Macedonia	Yes	No	Yes	Yes **	Yes	No
Moldova	Yes	No	Yes	No	Yes	No
Romania	No	Yes	No	No	No	No
Russia	No	Yes	Yes	Yes *	Yes	No
Slovakia	No	Yes	Yes	No	Yes	No
Turkey	No	Yes	Yes	Yes **	Yes	No
Ukraine	No	Yes	Yes	No	Yes	No

* for certain ages, refer to the country section

** for certain treatments

**Table 4:** Summary of data on dental personnel and who provides oral health care for the 0-16 year- olds in the ECC reported by participants in the workshop

Countries	Dental Personnel				
	General Dentists	Children’s Dentists	Orthodontists	Dental Hygienists	% of Dentists working with Dental Nurse
Albania	Yes	Yes	Yes	No	Unclear
Armenia	No	Yes	Yes	No	100 %
Belarus	No	Yes	Yes	No	100 %
Bulgaria	Yes	Yes	Yes	No	40 %
Croatia	Yes	Yes	Yes	No	100 %
Czech Republic	Yes	No	Yes	Yes	100 %
Georgia	No	Yes	Yes	No	25 %
Greece	Yes	No	Yes	No	Unclear
Hungary	Yes	Yes	No	Yes	100 %
Latvia	Yes	Yes	Yes	Yes	100 %
Lithuania	Yes	Yes	No	No	50 %
Macedonia	Yes	Yes	Yes	No	100 %
Moldova	Yes	Yes	Yes	No	20 %
Romania	Yes	Yes	Yes	No	<20 %
Russia	Yes	Yes	Yes	No	Unclear
Slovakia	Yes	Yes	Yes	Yes	100 %
Turkey	Yes	Yes	Yes	No	<10 %
Ukraine	No	Yes	Yes	Yes	<90 %

**Table 5:** Dental caries experience among 6,12 and 15 year-olds in Belarus in 2008

Age, years	Prevalence, %	Level	Dental caries experience			
			dmft	DMFT	DMFT+dmft	Level
6	80,02	High	4,35	0,07	4,42	medium
12	69,42	medium	0,06	2,14	2,20	medium
15	80,63	High	0	3,38	3,38	medium

**Table 6:** Oral hygiene status among 6,12 and 15 year-olds in Belarus in 2008

	Oral Hygiene Status (OHI-S or PlI)	
Age	mean	interpretation
6	1,00	satisfactory
12	1,05	satisfactory
15	0,96	satisfactory

**Table 7:** Data on oral health for 5, 12, 18 year-olds in urban and rural areas in Bulgaria in 2010

indices	urban	rural	total
dmf 5-6 year	3,32	4,05	3,69
DMF 12 years	2,69	3,37	3,03
DMF 18 years	5,98	6,51	6,25
% free of caries 5-6	31,73 %	26,0 %	28,87 %
% free of caries 12	25,82 %	16,81 %	21,31 %
% free of caries 18	10,17 %	6,47 %	8,31 %
Mean plaque scores 5-6 years	1,03	1,13	1,08
Mean plaque scores 12 years	1,73	1,75	1,74
Mean plaque scores 18 years	1,63	1,73	1,67
Fluorosis 5-6 years	3,30	2,36	2,83
Fluorosis 12 years	10,11	6,15	8,13
Fluorosis 18 years	3,55	2,54	3,04
CPITN = 0 at 18 years	39,07	30,39	34,73

**Table 8:** Data on malocclusion for 5, 12, 18 year-olds in Bulgaria in 2010

Malocclusion (%)	none	first degree	second degree
5-6 year old	60,26	27,37	12,37
12 year old	29,07	39,5	31,43
18 year old	35,74	39,70	24,54

**Table 9:** Specialists in Orthodontics and Children’s Dentistry with Croatian Health Insurance Contracts in 2013

Specialists in orthodontics - with contracts with Croatian Health Insurance Fund		
Hospitals	5,2	
Health care centers	22,7	
Policlinics	34,88 (21 private, 13,88 state)	
Private offices	69	
Specialists in children’s dentistry - with the contracts with Croatian Health Insurance Fund		
	Number of teams	Number of specialists
Clinical Hospital Center ZAGREB	5,9	7
Clinical Hospital Center RIJEKA	1	2
Dental policlinic ZAGREB	2	2
The Croatian Health Insurance Fund - plan to enlarge the teams of children’ dentistry dentistry		
Region	Number teams	
Osječko-baranjska	3	
Primorsko-goranska	2	
Splitsko-dalmatinska	4	
Grad Zagreb	8	
Total	17

**Table 10:** Caries experience among the 5, 12 and 15 year-olds in Greece (2001-2005)

	5- year -olds	12- year- olds	15- years- olds
DMFT	1,77	2,05	3,19
DT	1,54	1,15	1,43
MT	0,006	0,02	0,05
FT	0,24	0,97	1,81
% without caries	57,2	37,1	28,9

**Table 11:** Oral hygiene index among the 5, 12 and 15 year-olds in Greece (2001-2005)

	Mean scores (indices of oral hygiene)			Oral hygiene (OHI levels)		
	DI-s	CI-s	OHI-s	Good	Medium	Bad
5 -year-olds	0,54	-	-	58,5	39,3	2,2
12 -year-olds	0,92	-	-	21,5	75,0	3,5
15-year-olds	0,74	-	-	37,2	61,4	1,4

Additional file 1 (cited in the original article as Fig. 1) is a questionnaire that was sent to all delegates at the workshop.

## Additional file

Additional file 1: The pre-conference questionnaire. (DOCX 14 kb)
