# Supplementary material for: Erratum to: Proceedings of a workshop, held in Constanta, Romania on 22 May 2014, on Oral Health of Children in the Central and Eastern European Countries in the context of the current economic crisis
Source: BMC Oral Health. 2016 Sep 15;16:96. doi: 10.1186/s12903-016-0269-x (PMC5025609; doi:10.1186/s12903-016-0269-x)
Supplement: Additional file 1: — The pre-conference questionnaire. (DOCX 14 kb) [file 12903_2016_269_MOESM1_ESM.docx]

**Additional file 1. The pre-conference questionnaire**

Please include answers to the following questions on the provision of oral health care to children (those aged up to 16 years of age).

Epidemiology - (The Need for Oral Health Care)

1. In which year was the last national study of the oral health of children in your country performed?

2. Which ages of children were examined?

3. What were its main results e.g. National mean DMFT figures, oral cleanliness, need for orthodontics, number of 16 year olds with traumatized anterior teeth?

Please bring a copy of the published results of this study or of these studies either as a published paper or a Government Report.

4. If there has been no national study of the oral health of children in your country, have there been regional or local studies? If so please give brief details of the results and bring the published results with you (published paper or a Government report).

Prevention

5. What programs are there in your country to prevent oral diseases in children?

6. Are they national or regional or local or all three?

7. Where do they take place - in schools or in dental offices/cabinet/clinics or elsewhere?

Treatment and Payment

8. Is there free oral treatment for all children up to the age of 16 years for all types of dentistry, extractions, fillings, crowns, scaling and polishing, orthodontics, fixed and removable prostheses, etc?

9. Or is there free oral treatment for all children up to the age of 16 years for only some items of treatment? If so please list which ones.

10. Who provides free treatment? Is it only in public clinics or is it in public clinics and private offices/cabinet/clinics.

Dental Personnel

11. Who provides oral health care for children in your country?

12. Is it only general dentists or general dentists and specialists in children’s' dentistry and orthodontists.

13. What percentage of dentists employ dental nurses (chair-side assistants) to help them?

14. What tasks do such dental nurses perform?

15. Are dental hygienists trained and employed in your country? If not why not?

Uptake of Oral Health Care

16. In your country, what percentage of children aged 16 years or less visited a dentist in the last 12 months?

17. Are there any parts of your country where it is difficult or impossible to visit a dentist?

18. If so why?

Other Considerations

19. How has the economic situation in the last 6 years influenced the provision of oral health care in your country?

20. In your opinion, at present, in your country is the oral health of children aged up to 16 years improving or deteriorating?

21. What needs doing to improve this situation?
